# A Retrospective Molecular Epidemiology Study: Emergence and dissemination of Azole-Resistant *Candida parapsilosis* Sensu Stricto in Southern Brazil

**DOI:** 10.1007/s11046-026-01095-0

**Published:** 2026-08-07

**Authors:** Luiza Souza Rodrigues, Sabrina da Conceição Barbosa, Ana Paula Santiago Chacon, Adriele Celine Siqueira, Gisele Aparecida Bernardi, Marcelo Pillonetto, Vania Aparecida Vicente, Lavinia Nery Villa Stangler Arend, Terezinha Inez Estivalet Svidzinski, Libera Maria Dalla-Costa

**Affiliations:** 1 Paraná State Public Health Laboratory (Lacen PR), Sebastiana Santana Fraga, 1395, Guatupê, São José dos Pinhais, PR CEP 83060-500 Brazil; 2 Faculdades Pequeno Príncipe, Av. Iguaçu, 333, Rebouças, Curitiba, PR CEP 80230-020 Brazil; 3 Instituto de Pesquisa Pelé Pequeno Príncipe, Av. Silva Jardim, 1632, Água Verde, Curitiba, PR CEP 80250-060 Brazil; 4 Department of Basic Pathology, Federal University of Paraná, Av. Cel. Francisco H. dos Santos, 100, Jardim da Américas, Curitiba, PR CEP 81531-980 Brazil; 5 Microbiological Collection of Paraná Network-Taxonline (CMRP/Taxonline), Department of Basic Pathology, Federal University of Paraná State, Av. Cel. Francisco H. dos Santos, 100, Jardim das Américas, Curitiba, PR CEP 81531-980 Brazil; 6 Universidade Estadual de Maringá, Av. Colombo, 5790, Jardim Universitário, Maringá, PR CEP 87020-900 Brazil

**Keywords:** *Candida parapsilosis*, Fluconazole-resistant, Invasive candidiasis, Candidemia, Microsatellite, Genome

## Abstract

*Candida parapsilosis* sensu stricto has emerged as a major cause of healthcare-associated candidemia, with increasing reports of fluconazole resistance worldwide. This resistance is most commonly associated with amino acid substitutions in Erg11p, particularly Y132F; however, regional variation in resistance mechanisms and clonal dissemination remains not fully understood. This study investigated the molecular epidemiology and genomic landscape of fluconazole-resistant *C. parapsilosis* in Paraná, Southern Brazil, over a 10-year period. A total of 54 resistant isolates identified between 2014 and 2024 were retrospectively analyzed; 51 viable isolates were confirmed by species identification, antifungal susceptibility testing, and microsatellite genotyping. A subset of 18 representative isolates underwent whole-genome sequencing for phylogenomic analysis. Variants in genes associated with azole resistance, including *ERG11*, *MRR1*, *TAC1*, *CDR1*, *MDR1*, *ERG3*, and *ERG6*, were characterized. Fluconazole resistance emerged in 2018 and increased thereafter. Phylogenomic analysis revealed two geographically structured lineages: a dominant clone circulating across multiple healthcare institutions in the Curitiba metropolitan area and a second lineage restricted to the northern region of the state. The dominant clone showed minimal genomic diversity (0–5 SNPs), consistent with long-term persistence. A conserved resistance profile was observed, with 17 of 18 sequenced isolates harboring the K143R substitution in Erg11p, while all retained the wild-type residue at position 132. The single isolate lacking *ERG11* mutations carried a novel C849Y substitution in the Mrr1p regulator. These findings demonstrate the emergence and persistence of a highly clonal fluconazole-resistant *C. parapsilosis* lineage in Southern Brazil, characterized by a conserved K143R substitution independent of Y132F. The detection of closely related isolates across different hospitals underscores the need for regional genomic surveillance of hospital-adapted fungal pathogens.

**Supplementary Information:**

The online version contains supplementary material available at https://doi.org/10.1007/s11046-026-01095-0.

## Introduction

Invasive candidiasis (IC) remains a major cause of morbidity and mortality among critically ill patients, particularly those admitted to intensive care units (ICUs) [1, 2]. Over the past decade, a global epidemiological shift has been observed, with an increasing incidence of non-*albicans Candida* species [3]. Among these, *Candida parapsilosis* sensu stricto has emerged as a leading cause of candidemia in several geographic regions, including Latin America [4, 5]. *Candida parapsilosis* exhibits a remarkable ability to adhere to intravascular devices, persist on healthcare workers’ hands, and form biofilms, thereby facilitating nosocomial transmission [6, 7]. Although historically highly susceptible to azoles, increasing rates of fluconazole resistance have been reported worldwide [8, 9].

The COVID-19 pandemic placed unprecedented strain on healthcare systems, leading to prolonged ICU stays, widespread use of invasive devices, increased empirical antifungal therapy, and disruptions in infection prevention and control practices [10]. These factors may have contributed to the emergence and selection of fluconazole-resistant *C. parapsilosis* (FRCP) lineages, while simultaneously facilitating their spread within healthcare settings [9]. Indeed, recent studies have reported intra-hospital dissemination of FRCP clones temporally associated with the pandemic period [11–13].

FRCP is most associated with mutations in the *ERG11* gene, particularly Y132F and K143R substitutions, and has been frequently linked to intra-hospital clonal expansion, supporting transmission-driven dissemination [13–16]. However, most available data derive from single-center or outbreak-focused investigations, and long-term, multicenter surveillance studies across defined geographic regions remain scarce [16, 17].

In this context, we conducted a retrospective, statewide, laboratory-based epidemiological study of FRCP isolates recovered in Paraná, Brazil, between 2014 and 2024, with the first resistant isolates identified in 2018. The aim of this study was to investigate the temporal emergence, inter-hospital dissemination, and molecular epidemiology of these isolates using microsatellite typing and whole-genome sequencing (WGS).

## Material and Methods

### Isolates and Clinical Setting

We conducted a retrospective laboratory-based study including all non-duplicate clinical *Candida parapsilosis* sensu stricto isolates (one isolate per patient) reported as fluconazole-resistant between January 2014 and December 2024 by the Central Laboratory of the State of Paraná (LACEN-PR), the public reference laboratory in Southern Brazil. Data were retrieved from the state laboratory information system, which integrates routine diagnostic results from public healthcare facilities throughout Paraná. A total of 54 fluconazole-resistant isolates originating from seven public hospitals located in different regions of Paraná were retrieved from the LACEN strain collection and included in the study. Stored isolates were subcultured to assess viability and purity on chromogenic *Candida* agar, and species identification was confirmed by matrix-assisted laser desorption/ionization time-of-flight mass spectrometry (MALDI-TOF MS; bioMérieux, Marcy-l'Étoile, France) according to the manufacturer's instructions. Isolates that could not be recovered after storage were excluded of the subsequent analyses. This study was approved by the Institutional Review Board (IRB) of the participating center (IRB #6.481.883). We ensured the anonymity of the patients involved.

### Antifungal Susceptibility Testing

Antifungal susceptibility testing was performed using the commercial broth microdilution system MICRONAUT-AM (Merlin Diagnostika, Bornheim, Germany), according to the manufacturer's instructions. Minimum inhibitory concentrations (MICs) were read visually after incubation according to the manufacturer's recommendations and interpreted following the Brazilian Committee on Antimicrobial Susceptibility Testing (BrCAST) version 12.1 (2026), harmonized with the European Committee on Antimicrobial Susceptibility Testing (EUCAST) clinical breakpoints. The following clinical breakpoints were applied: fluconazole, susceptible (S) ≤ 2 µg/mL and resistant (R) > 4 µg/mL; voriconazole, S ≤ 0.125 µg/mL and R > 0.25 µg/mL; amphotericin B, S ≤ 1 µg/mL and R > 1 µg/mL; and anidulafungin and micafungin, S ≤ 4 µg/mL and R > 4 µg/mL [18]. Quality control was performed using *Candida parapsilosis* ATCC 22019 and *Candida krusei* ATCC 6258.

### Microsatellite Typing

Genomic DNA was extracted from pure cultures grown on Sabouraud dextrose agar. A 10-µL loopful of pure colony material was suspended in 300 µL of LB5 buffer (Loccus, Cotia, São Paulo, Brazil) and subjected to mechanical disruption using a tissue homogenizer (L-BEADER 6, Loccus, Cotia, São Paulo, Brazil). DNA extraction was subsequently performed using the automated TANBead Maelstrom™ 9600 platform (TANBead, Taoyuan, Taiwan) with the Extracta Kit Gold (Loccus, Cotia, São Paulo, Brazil), according to the manufacturer’s instructions.

Genotyping of all *C. parapsilosis* sensu stricto isolates and the reference strain *C. parapsilosis* ATCC 22019 was performed using PCR amplification of eight polymorphic microsatellite markers as previously described [19]. PCR amplicons were analyzed by automated electrophoresis using the TapeStation system (Agilent Technologies, Santa Clara, California, United States) for fragment size determination and allelic profile analysis.

Allelic profiles were converted into binary matrices, where “1” indicated presence and “0” absence of a given allele. Isolates sharing identical allelic profiles across all eight markers were considered genetically indistinguishable (clonal) [20]. Three of the 54 isolates could not be successfully genotyped.

### Whole-Genome Sequencing

A subset of 18 FRCP isolates was selected for whole-genome sequencing (WGS) from the 51 viable isolates that underwent microsatellite genotyping. The selection was designed to represent the temporal distribution of cases, the geographic distribution across participating hospitals, and all microsatellite genotypes (clones) identified in the study, thereby reflecting the overall epidemiological scenario observed during the study period. Genomic DNA libraries were prepared and sequenced on the Illumina MiSeq platform (Illumina, San Diego, CA, USA) using the MiSeq Reagent Kit v3 (2 × 300 bp paired-end reads). Read data were submitted to the Sequence Read Archive (SRA) database from the National Center for Biotechnology Information (NCBI), BioProject accession PRJNA1430745.

Bioinformatic processing was performed using the MycoSNP-nf v1.4 pipeline (https://github.com/CDCgov/mycosnp-nf, accessed March 2026). Raw reads were quality-trimmed with FaQCs and aligned to the *C. parapsilosis* CDC317 reference genome (GCA_000182765.2) using BWA-mem. Single-nucleotide polymorphisms (SNPs) were called and filtered using a minimum quality score of 30 and a minimum depth of coverage of 10x. Repetitive regions in the reference genome were identified via self-alignment with *MUMmer* v3.23 and masked to ensure high-confidence variant calling. A high-resolution maximum-likelihood phylogenetic tree was reconstructed from the core-SNP alignment using *IQ-TREE* v2.x. The best-fit nucleotide substitution model was selected by ModelFinder with ascertainment bias correction (+ ASC). Branch support was estimated using Ultrafast Bootstrap with 1,000 replicates, and the final tree was visualized in *iTOL* v6. Pairwise SNP distances were calculated using *snp-dists* v1.2.0 to assess genetic relatedness. Pairwise SNP distances were used to assess genetic relatedness among isolates. Isolates were considered genetically related or clonal when separated by approximately 12 SNPs, based on established thresholds for *Candida* outbreaks [21]. Phylogenetic clades were defined by robust clustering supported by large genomic distances, typically involving tens of thousands of SNP differences between clades, whereas isolates within the same clade were highly related, usually differing by fewer than 100 SNPs [22].

### Molecular Characterization of Azole Resistance-Associated Genes

To investigate the molecular basis of azole resistance, the full-length sequences of key resistance-associated genes, including *ERG11* (CPAR2_303740), *TAC1*, *MRR1*, *CDR1* (CPAR2_405290), *MDR1* (CPAR2_301760), *ERG3* (CPAR2_105550), and *ERG6* (CPAR2_405010), were analyzed for all 18 clinical isolates using a genome assembly and variant-based approach.

Raw sequencing reads were initially assessed for quality using *fastQC* v0.12.1 and subsequently trimmed to remove low-quality bases sequences using *Trimmomatic* v0.39. High-quality reads were de novo assembled using *SPAdes* v3.14.1*.* All contigs with a size <  = 500 bp were excluded, and the quality of the assemblies was assessed using *QUAST* v5.2.0*.* Gene annotation was performed using *Liftoff* v1.6.3 employing the genome of the reference strain *Candida parapsilosis* CDC317 as a reference.

Target genes were identified within the annotated assemblies, and genomic regions corresponding to each locus were defined based on the CDC317 reference genome (GenBank: GCA_000182765.2). To ensure consistency across isolates, consensus sequences were reconstructed for each gene using a reference-guided approach. Briefly, variant call files (VCF) were applied to the CDC317 reference genome using the *bcftools consensus* command, generating isolate-specific consensus sequences. Gene-specific regions were then extracted using *samtools faidx* based on curated genomic coordinates. It is important to note that  although CDC317 is the standard genomic reference for this species, it is genotypically characterized as azole-resistant, harboring the resistance-associated substitution Y132F in the *ERG11* gene.

The resulting nucleotide sequences were translated in silico to obtain the corresponding protein sequences (Erg11p) using the ExPASy Translate tool (https://web.expasy.org/translate/; accessed March 2026). Multiple sequence alignments of the predicted protein sequences were performed using Clustal Omega v1.2.4 with default parameters. The resulting alignments were used to identify amino acid substitutions relative to the reference sequence. Multiple sequence alignments of Erg11p were performed using ESPript v3.0 (https://espript.ibcp.fr/ESPript/cgi-bin/ESPript.cgi; accessed March 2026), with a focus on identifying non-synonymous substitutions relative to the wild-type Erg11p.

In addition to the targeted analysis of azole resistance-associated genes, the predicted proteomes of all isolates were screened for additional antimicrobial resistance determinants using AMRFinderPlus (NCBI Antimicrobial Resistance Gene Finder; Galaxy Version 3.12.8 + galaxy0) via the Galaxy Europe platform (accessed March 2026), employing the curated Hidden Markov Model (HMM) library [23].

## Results

### Temporal Emergence and Clinical Distribution of FRCP

**Fig. 1:**
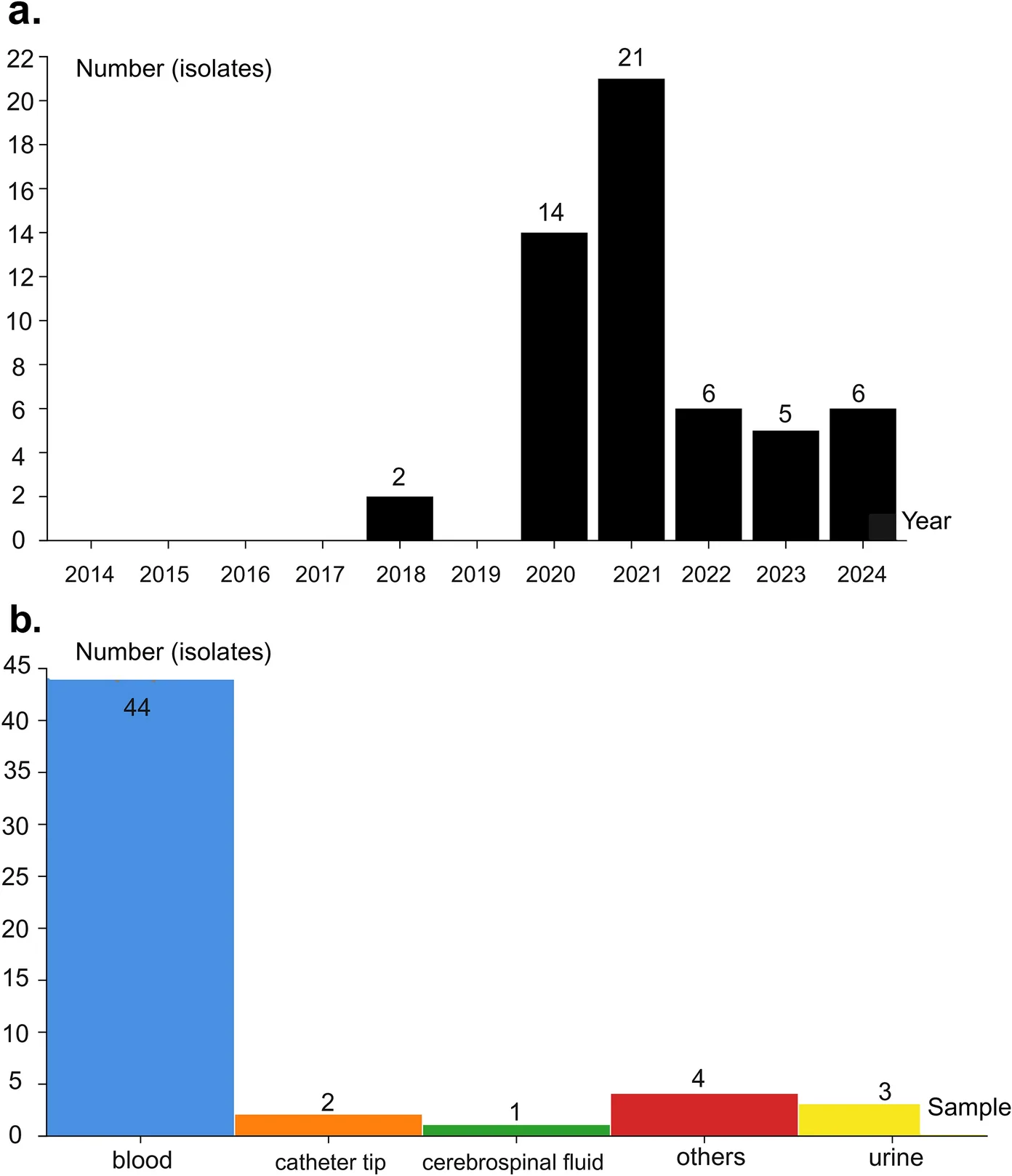
Temporal trend and clinical source distribution of FRCP isolates (n = 54) in Paraná, Brazil, 2014–2024. **a**. Annual number of FRCP isolates is identified by the state reference laboratory. **b**. Distribution of isolates according to clinical specimen type

A total of 54 FRCP isolates were identified between 2018 and 2024 through the state reference laboratory. The clinical, epidemiological, phenotypic, and microsatellite genotyping data for all isolates are summarized in Supplementary table S1. No fluconazole-resistant isolates were reported by LACEN between 2014 and 2017, and in 2019. After the initial detection in 2018, a marked increase in FRCP cases was observed in 2020 and 2021, with sustained circulation in subsequent years (Fig. 1a). Most FRCP isolates were recovered from bloodstream infections, with fewer isolates from urine, cerebrospinal fluid, catheter tips, and other clinical specimens (Fig. 1b).

### Antifungal Susceptibility Testing

Out of 54 fluconazole-resistant *C. parapsilosis* isolates retrieved for this study, 51 remained viable and were confirmed as resistant to fluconazole (MIC > 4 µg/mL). Among these, 53% (27/51) exhibited fluconazole MICs ≥ 32 µg/mL. For voriconazole, 54.9% (28/51) of isolates were categorized as susceptible (MIC ≤ 0.125 µg/mL), whereas 45.1% (23/51) were classified as susceptible, increased exposure (MIC = 0.25 µg/mL). All isolates remained susceptible to amphotericin B (MIC ≤ 1 µg/mL) and echinocandins (anidulafungin and micafungin; MIC ≤ 4 µg/mL) (Table 1 and Supplementary table S1).

**Table 1:** Minimal Inhibitory Concentration (MIC) distribution of antifungal agents against *Candida parapsilosis* clinical isolates (n = 51)

Antifungal agent	MIC (μg/mL)													
	< = 0.03	0.06	0.125	0.25	0.5	1	2	4	8	16	32	> 32	MIC50	MIC90
Fluconazole	–	–	–	–	–	–	–	–	3	21	11	16	32	> 32
Voriconazole	–	11	17	23		–	–	–	–	–	–	–	0.125	0.25
Micafungin	1	8	23	19	–	–	–	–	–	–	–	–	0.125	0.25
Anidulafungin	–	1	13	36	1	–	–	–	–	–	–	–	0.25	0.25
Amphotericin B	–	–	–	–	42	8	–	–	–	–	–	–	0.5	1

### Microsatellite Genotyping and Clonal Dissemination

**Fig. 2:**
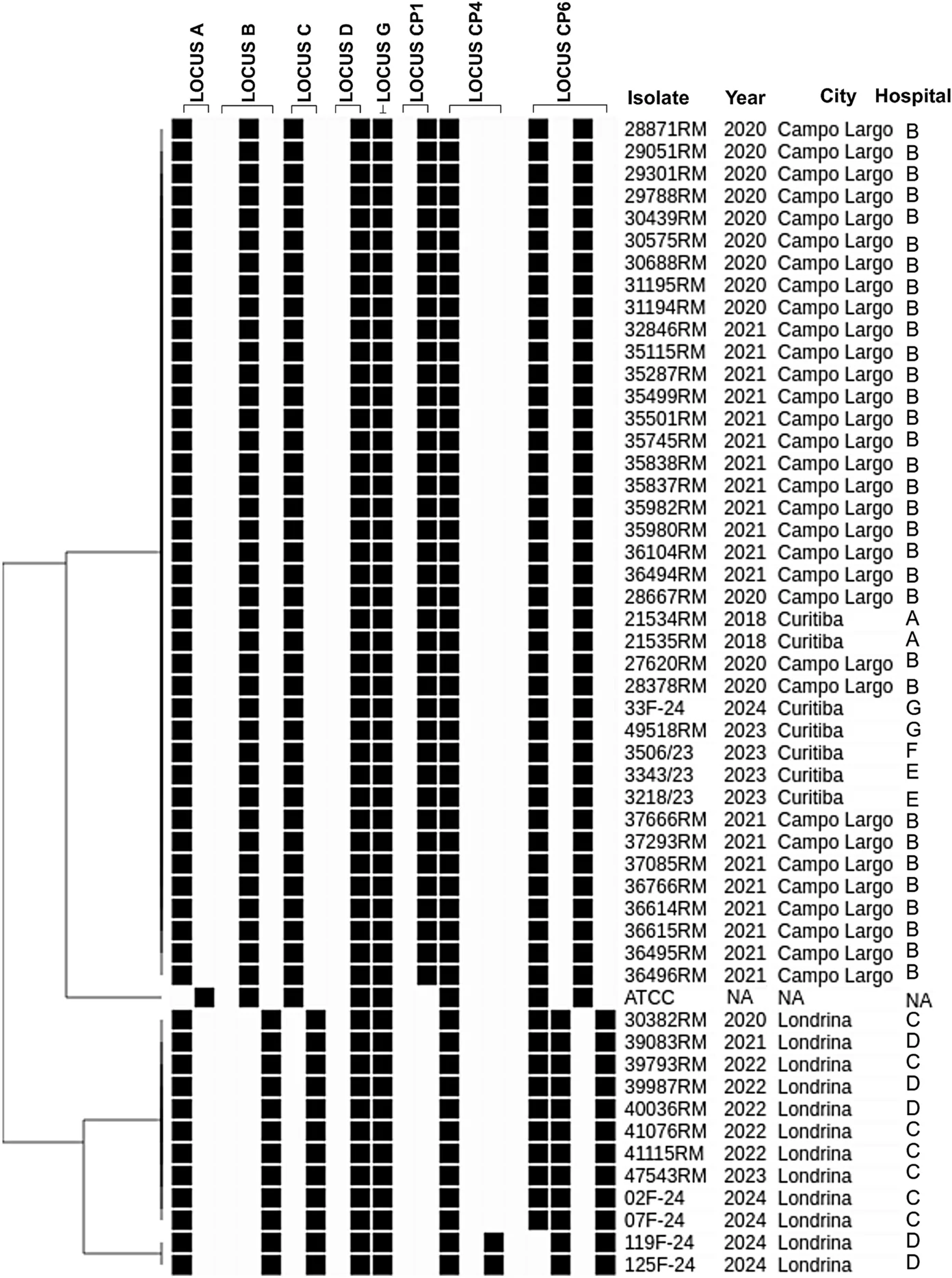
Microsatellite-based dendrogram showing genetic relatedness among FRCP isolates recovered in Paraná, Brazil (2018–2024). Black squares indicate the presence of an amplification product for a given allele. Columns are grouped according to the eight microsatellite loci (A, B, C, D, G, CP1, CP4, and CP6), with each column representing a distinct allele within the corresponding locus. Three endemic clones (1–3) were identified. Clone 1 was detected in isolates recovered from multiple healthcare institutions in the Curitiba metropolitan region and Campo Largo, whereas Clones 2 and 3 were restricted to Londrina. The hospital of origin and year of isolation are indicated alongside each isolate

Microsatellite genotyping was successfully performed for 51 viable FRCP isolates (see Fig. 2). Three circulating genotypes (designated clones 1, 2, and 3) were identified, in addition to the reference strain, *C. parapsilosis* ATCC 22019. Clone 1, the most frequent genotype, was detected in multiple hospitals and two cities between 2018 and 2024. Clone 2 was identified in two hospitals between 2020 and 2024. Clone 3 was restricted to a single hospital and was the least frequent genotype.

### Phylogenomic Analysis and High-Resolution Clustering

**Fig. 3:**
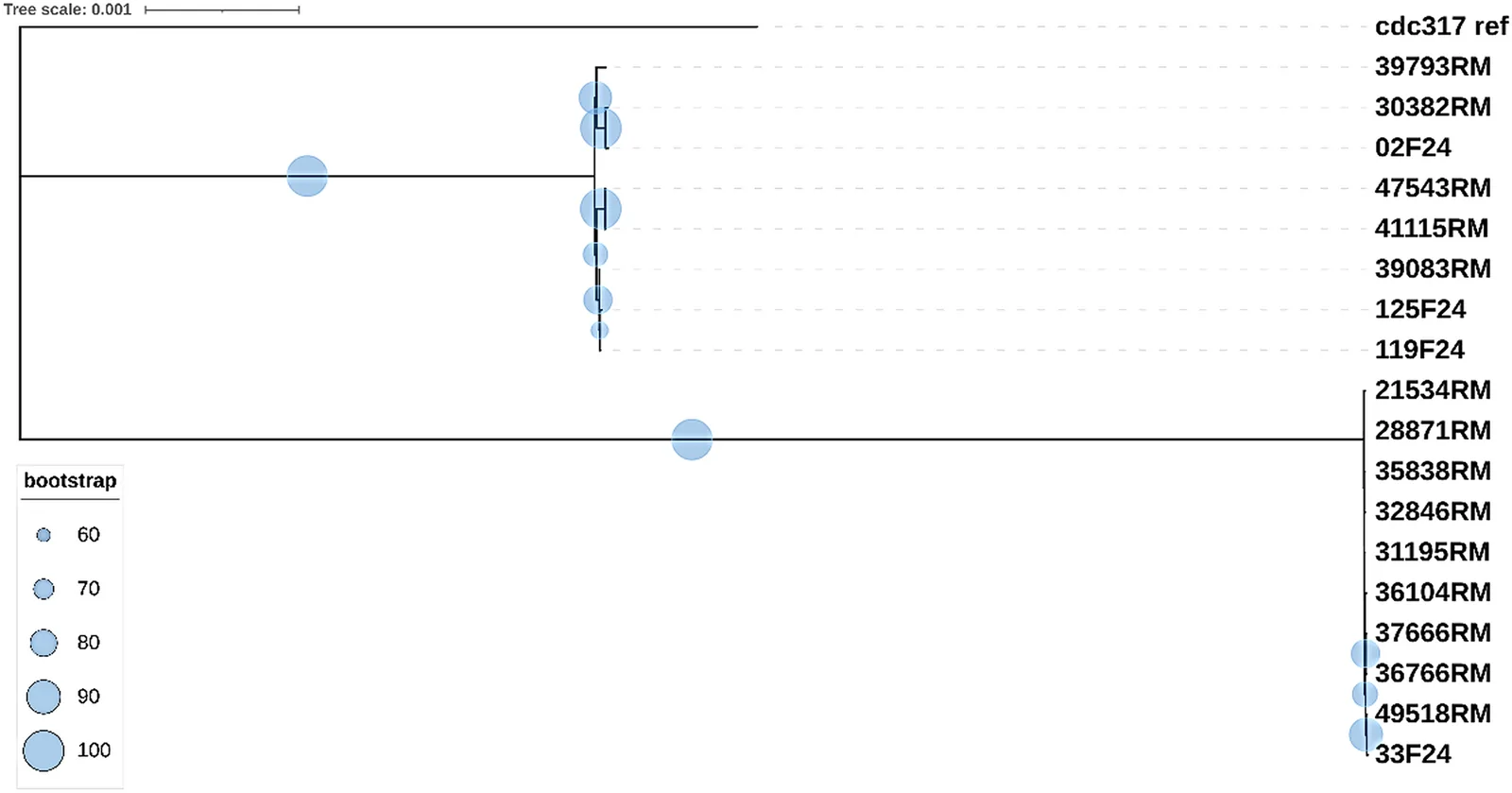
High-resolution phylogeny of *Candida parapsilosis* isolates. Maximum-likelihood phylogenetic tree visualized in iTOL. Nodes are annotated with bootstrap support values, with bubble sizes indicating confidence levels (60–100%). The tree highlights the clear divergence between the metropolitan cluster (bottom) and the northern cluster (top). Scale bar indicates nucleotide substitutions per site

Phylogenomic analysis was performed on 18 representative fluconazole-resistant *C. parapsilosis* isolates selected for whole-genome sequencing. Sequencing metrics, epidemiological metadata, and resistance-associated amino acid substitutions for all sequenced isolates are summarized in Supplementary table S2. The core-genome SNP analysis revealed high genetic relatedness among the isolates, providing superior resolution over microsatellite typing (Fig. 3). The pairwise SNP distance matrix confirmed a dominant clonal cluster (Clone 1) with extremely low genetic diversity (0 to 5 SNPs). Notably, isolates such as 28871RM and 31195RM from Hospital B exhibited zero SNP differences (Supplementary table S3). A second lineage, separated by approximately 1,850 SNPs, was identified primarily in the northern region of the state. While microsatellite typing previously divided this group into Clones 2 and 3, SNP analysis demonstrated they belong to the same genomic lineage; for instance, 119F24 (Hospital D) and 39083RM (Hospital D) differed by only 1 SNP, while 41115RM and 47543RM (Hospital C) were identical (0 SNPs). Within this second lineage, isolate 39793RM (Hospital C) showed a slightly higher degree of divergence, with a pairwise distance of approximately 20 SNPs from its closest relatives.

**Fig. 4:**
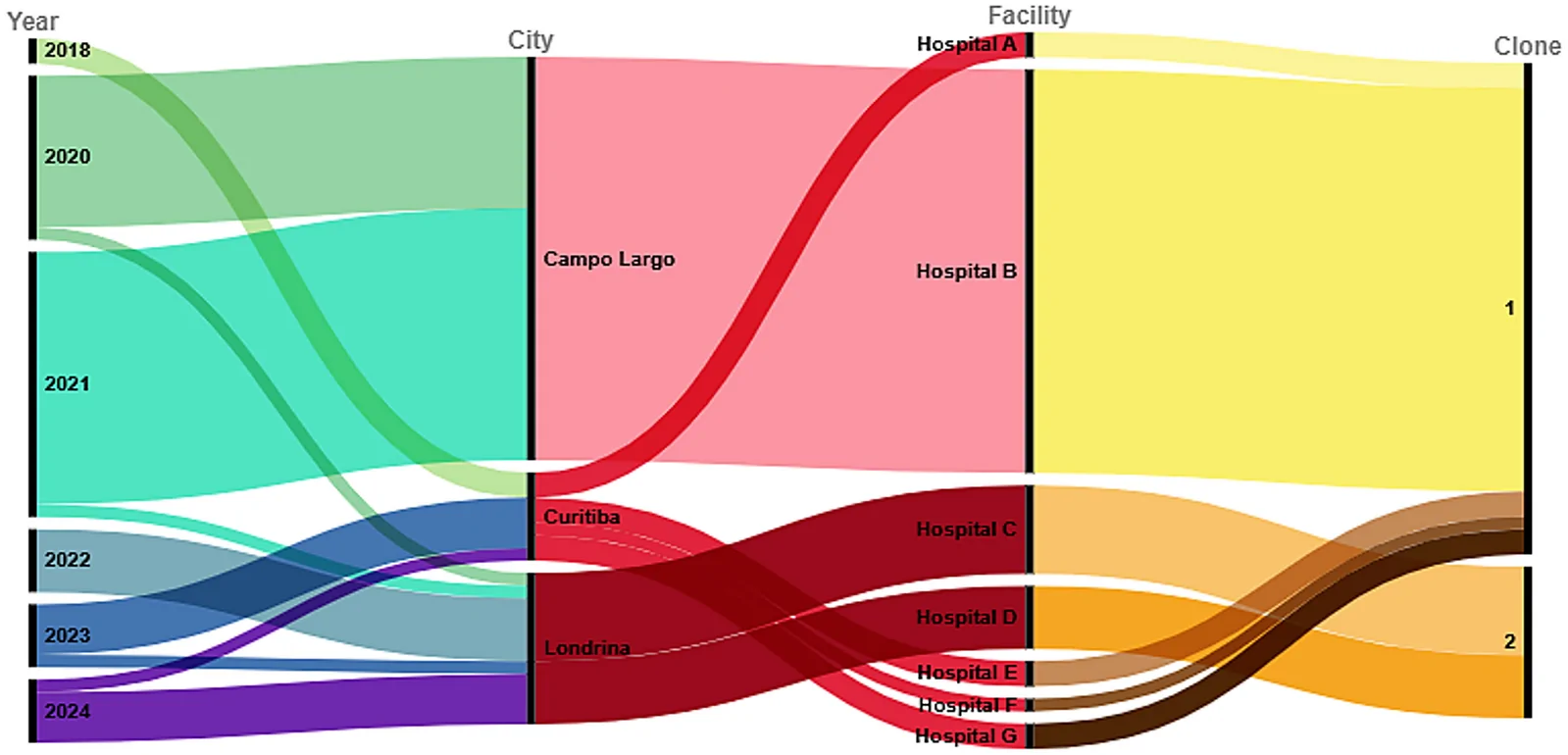
Spatiotemporal flow of *C. parapsilosis* clones across healthcare facilities in Paraná. Sankey diagram (generated via RAWGraphs) showing the distribution of isolates by year, city, and healthcare facility (Hospitals A–G). The flow thickness represents the number of isolates

Spatiotemporal analysis (Fig. 4) revealed the persistence of Clone 1 over a six-year period (2018–2024). This lineage first emerged in 2018 at Hospital A, followed by the identification of genetically related isolates at Hospital B between 2020 and 2021. Between 2023 and 2024, the clone was identified in Hospitals E, F, and G, indicating an ongoing inter-hospital dispersal within the Curitiba metropolitan area. In contrast, the second genomic lineage remained strictly localized to Hospitals C and D in Londrina throughout the study period.

### Molecular Characterization of Azole Resistance-Associated Genes

Analysis of Erg11p sequences in the 18 clinical isolates showed high homology with the *C. parapsilosis* CDC317 reference strain, with key differences identified at resistance-associated (hotspots) positions. Contrary to the CDC317 reference, which harbors the Y132F substitution, all 18 clinical isolates retained the wild-type tyrosine residue (Y132).

**Fig. 5:**
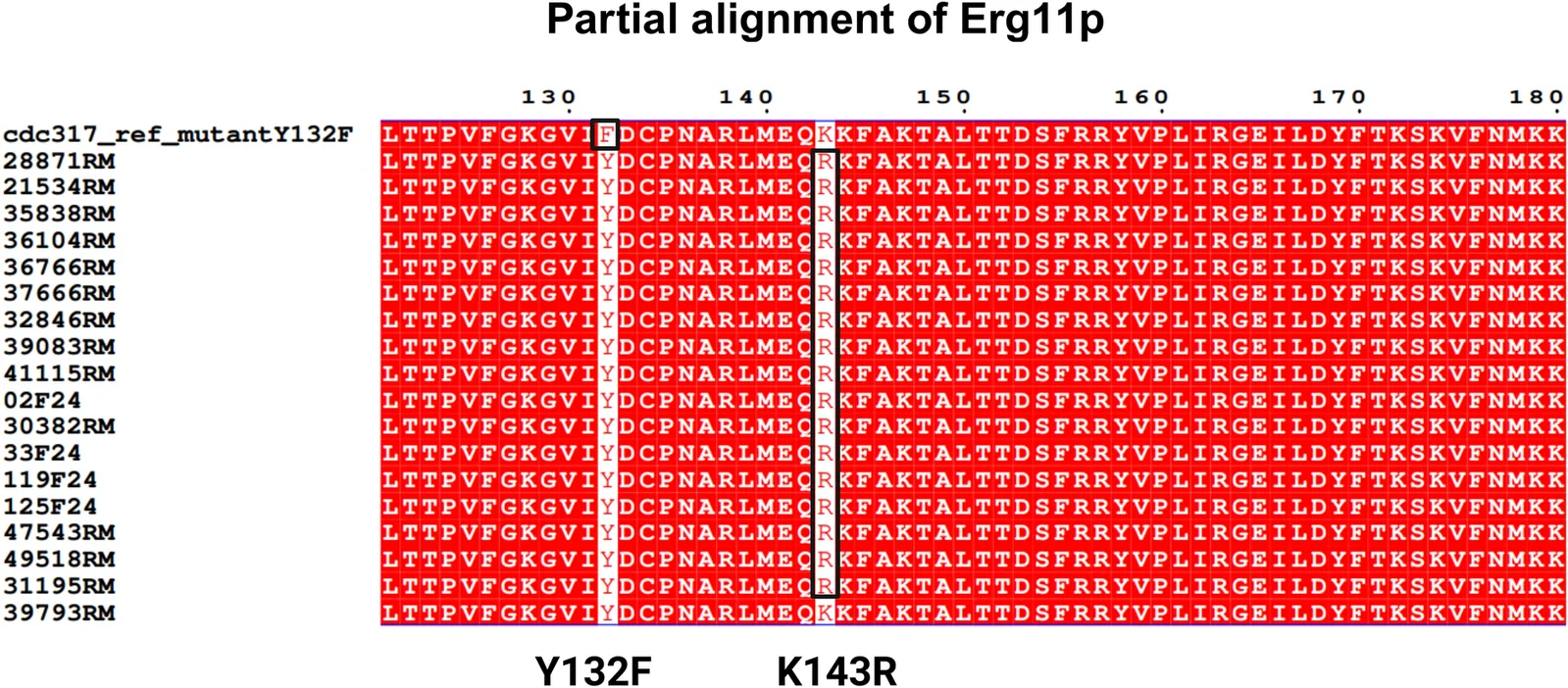
Multiple sequence alignment of partial Erg11p amino acid sequences from clinical isolates and the reference strain highlights substitutions at known azole resistance hotspots. Contrary to the reference strain, none of the isolates have the Y132F mutation. All isolates contain the K143R mutation, except for isolate 39793RM, which shows an absence of mutations at positions Y132 and K143

In contrast, a different pattern was observed at position 143. While the CDC317 reference carries the wild-type lysine (K143), 17 out of 18 isolates exhibited the K143R substitution, a well-known marker for azole resistance. Only one isolate (39793RM) matched the reference at this locus, retaining the wild-type K143 residue (Fig. 5). No other non-synonymous substitutions were found in the Erg11p protein in the sequences analyzed.

To further investigate the resistance mechanism in the *ERG11* wild-type isolate (39793RM), the *MRR1* regulatory gene was analyzed. This isolate harbored a novel C849Y amino acid substitution in Mrr1p. Additionally, several novel non-synonymous substitutions not previously described were identified across the collection, including Tac1p R208G, Erg3p H260Q, and the dual substitution Erg6p S208G + S304G. These variants were predominantly distributed according to clonal lineage (Supplementary table S2).

When using AMRFinderPlus to identify additional resistance determinants, no hits were found that corresponded to known or recently described determinants, including acetyltransferase domains associated with azole resistance.

## Discussion

The emergence and intra-state dispersal of FRCP represent an increasing concern within the mycological landscape of Southern Brazil. Notably, the number of FRCP isolates increased markedly between 2020 and 2021, simultaneous with the COVID-19 pandemic. Although the retrospective design of our study does not allow us to establish a causal relationship, this temporal pattern is consistent with reports from Brazil and other countries describing increased dissemination of fluconazole-resistant *C. parapsilosis* during the pandemic [11–13]. Overburdened healthcare systems, prolonged ICU stays, increased use of invasive devices, widespread empirical antifungal therapy, and challenges in maintaining infection prevention and control measures have all been proposed as factors contributing to the emergence and dissemination of resistant *C. parapsilosis* clones [10–13].

Our findings reveal a scenario of high clonal stability and long-term persistence, with Clone 1 circulating across at least five healthcare institutions in the Curitiba metropolitan area over a six-year period (2018–2024). This pattern of silent persistence mirrors long-term clonal transmission dynamics previously described, including reports of sustained dissemination over decades within single tertiary care centers in Turkey [24].

The detection of fluconazole resistance in Paraná was contemporary with the first reports of clonal outbreaks in Southeastern Brazil. Our earliest resistant isolate dates to 2018, coinciding with the detection of the Y132F amino acid change in São Paulo by Thomaz et al. [11]. It is important to note that, as this study is based on retrospective analysis rather than prospective surveillance, this date may underestimate the true onset of FRCP emergence. However, in contrast to the São Paulo clusters, where Y132F was identified as the primary and often exclusive resistance-associated mutation in the Erg11p, our isolates were characterized by the predominance of the K143R substitution in the absence of Y132F, suggesting a distinct regional evolutionary pathway [11].

While the Y132F substitution has been widely reported as the predominant mechanism of azole resistance in *C. parapsilosis*, alternative mutations may also play a significant role. Among these, the K143R substitution in Erg11p has been described in clinical isolates and outbreak settings and is associated with reduced azole susceptibility, either alone or in combination with additional mechanisms such as efflux pump upregulation mediated by transcriptional regulators. Notably, although reported internationally, to our knowledge, K143R has not been described as a predominant resistance-associated mutation in Brazilian cohorts, where Y132F remains the most frequent substitution. This underscores the complexity of resistance phenotypes in *C. parapsilosis* and supports the existence of multiple evolutionary pathways to antifungal resistance. In this context, the predominance of K143R in our isolates reinforces its relevance within specific genomic backgrounds [9, 21, 25–28].

A clear geographic partition was observed, with Clone 1 dominating in the Metropolitan Region of Curitiba, Southeastern Paraná, while a second, phylogenomically distinct lineage was identified in Northern Paraná. The deep evolutionary divergence between these two clusters, separated by nearly 2,000 SNPs, supports independent evolutionary trajectories within the state rather than a single transmission event.

While microsatellite markers provided reliable preliminary clustering, the higher resolution of WGS refined this classification. Isolates previously assigned to distinct groups (Clone 2 and Clone 3) by microsatellite analysis differed by a maximum of only 25 SNPs, indicating they belong to a single genomic lineage. Within Clone 1, WGS confirmed strict clonality, with zero SNP differences observed between isolates such as 28871RM and 31195RM. This degree of genomic conservation across different healthcare institutions underscores the persistence of these resistant lineages even in the absence of documented epidemiological links.

Although the absence of detailed clinical metadata precludes reconstruction of direct transmission routes, the remarkable genomic stability observed among closely related isolates recovered from different hospitals over several years is consistent with the persistence and regional circulation of these lineages. Notably, the northern cluster exhibits internal genomic diversity; isolate 39793RM lacks non-synonymous mutations in the *ERG11* gene despite its resistant phenotype and shows a slightly higher SNP distance compared to closely related isolates. Further analysis revealed that the isolate harbors a novel C849Y substitution in the Mrr1p transcriptional regulator. Although this specific variant has not been previously described, it is located within the C-terminal activation domain, a known hotspot for gain-of-function mutations in this transcription factor; such mutations can result in the constitutive hyperactivation of efflux pumps, such as Mdr1p and Cdr1p, leading to resistance [28, 29]. This finding suggests ongoing microevolution within this lineage and indicates that azole resistance in this genomic background is not exclusively dependent on *ERG11* alterations but also involves increased drug efflux driven by regulatory mutations. However, the contribution of the C849Y substitution to the resistant phenotype remains to be functionally validated.

In addition to the established resistance markers, our analysis identified several non-synonymous substitutions that appear to be lineage specific. Two non-synonymous substitutions that had not been previously described were found in Tac1p: the R208G + G979D substitution and the dual S208G + S304G alterations in Erg6p, which had previously been described in susceptible and resistant isolates [30], were consistently found across Clone 1 isolates. Furthermore, the H260Q variant in Erg3p and the K1496E variant in Cdr1p, both of which had not been previously described, were each restricted to a single isolate (119F24 and 125F24, respectively). Further functional characterization is required to determine the potential synergistic role of these variants in the resistance landscape.

Historically, *C. parapsilosis* has been recognized for its propensity to cause healthcare-associated outbreaks regardless of antifungal susceptibility profiles [6, 7]. However, recent epidemiological studies have shown that fluconazole-resistant populations exhibit reduced genetic diversity and a highly clonal structure compared with susceptible isolates. This global trend of clonal expansion reflects the successful adaptation of specific resistant genotypes to hospital environments [11–14, 16, 17, 21, 22, 24, 25, 31].

Although this study provides a comprehensive genomic characterization of fluconazole-resistant *C. parapsilosis* isolates, contemporaneous fluconazole-susceptible isolates were not included in the whole-genome sequencing analysis. Therefore, while the association of the K143R substitution in Erg11p with azole resistance is well established, the contribution of the additional amino acid substitutions identified in this study, particularly those not previously described, remains uncertain. These variants should therefore be interpreted cautiously until their role in antifungal resistance has been further elucidated.

At the molecular level, our findings demonstrate a clear predominance of the K143R substitution in Erg11p in the absence of the widely reported Y132F substitution. This distinct mutational profile suggests that azole resistance in the Paraná isolates has evolved through a regional evolutionary trajectory different from that described in other settings [25]. These findings reinforce the importance of localized genomic surveillance to uncover region-specific evolutionary patterns. Elucidating how these distinct mutational profiles shape resistance phenotypes is essential not only for tracking clonal dissemination but also for improving our understanding of the evolution of antifungal resistance in hospital environments.

In conclusion, this study demonstrates the emergence of a highly clonal FRCP lineage in Southern Brazil. This spread is predominantly associated with the K143R substitution in Erg11p, notably in the absence of the Y132F mutation. However, the identification of a resistant isolate with a wild-type *ERG11* genotype reveals a more complex and adaptive genetic landscape. The detection of the novel Mrr1p C849Y substitution in this specific isolate, along with other lineage-specific variants in Tac1p, Erg3p, and Erg6p, underscores the critical role of WGS in identifying the full evolutionary spectrum of alternative resistance mechanisms. These findings highlight a distinct regional resistance pattern, and emphasize  the need for continuous genomic surveillance to monitor the spread and evolution of these resistant clones.

## Supplementary Information

Below is the link to the electronic supplementary material.

Supplementary file 1 (XLSX 62 KB)

Supplementary file 2 (XLSX 34 KB)

Supplementary file 3 (XLSX 53 KB)

## Data Availability

The datasets generated for this study can be found in the NCBI site under the BioProject accession code PRJNA1430745 https://www.ncbi.nlm.nih.gov/bioproject/PRJNA1430745

## Abbreviations

BrCAST: Brazilian Committee on Antimicrobial Susceptibility Testing
LACEN-PR: Central Laboratory of the State of Paraná
EUCAST: European Committee on Antimicrobial Susceptibility Testing
FRCP: Fluconazole-resistant *Candida parapsilosis*
ICUs: Intensive care units
IC: Invasive candidiasis
IRB: Institutional Review Board
MALDI-TOF MS: Matrix-assisted laser desorption/ionization time-of-flight mass spectrometry
MICs: Minimum inhibitory concentrations
NCBI: National Center for Biotechnology Information
SRA: Sequence Read Archive
SNPs: Single-nucleotide polymorphisms
WGS: Whole-genome sequencing

## References

[1] Cornely OA, Sprute R, Bassetti M, et al. Global guideline for the diagnosis and management of candidiasis: an initiative of the ECMM in cooperation with ISHAM and ASM. Lancet Infect Dis. 2025;25(5):e280–93. 10.1016/S1473-3099(24)00749-7. 39956121

[2] Soriano A, Honore PM, Puerta-Alcalde P, et al. Invasive candidiasis: current clinical challenges and unmet needs in adult populations. J Antimicrob Chemother. 2023;78(7):1569–85. 10.1093/jac/dkad139. PMC10320127 37220664

[3] Bongomin F, Gago S, Oladele RO, Denning DW. Global and multi-national prevalence of fungal diseases—estimate precision. J Fungi. 2017. 10.3390/jof3040057. PMC5753159 29371573

[4] Nucci M, Queiroz-Telles F, Alvarado-Matute T, et al. Epidemiology of candidemia in Latin America: a laboratory-based survey. PLoS ONE. 2013. 10.1371/journal.pone.0059373. PMC3601956 23527176

[5] Bays DJ, Jenkins EN, Lyman M, et al. Epidemiology of invasive candidiasis. Clin Epidemiol. 2024;16:549–66. 10.2147/CLEP.S459600. PMC11366240 39219747

[6] Branco J, Miranda IM, Rodrigues AG. *Candida parapsilosis* virulence and antifungal resistance mechanisms: a comprehensive review of key determinants. J Fungi. 2023. 10.3390/jof9010080. PMC9862255 36675901

[7] Tóth R, Nosek J, Mora-Montes HM, et al. *Candida parapsilosis*: from genes to the bedside. Clin Microbiol Rev. 2019. 10.1128/CMR.00111-18. PMC6431126 30814115

[8] Guinea J, Escribano P, Cadeau M, Lombardi L, Morio F. Emerging antifungal resistance in *Candida parapsilosis*: the end of the innocence. npj Antimicrob Resist. 2025. 10.1038/s44259-025-00173-5. PMC12708646 41402477

[9] Escribano P, Guinea J. Fluconazole-resistant *Candida parapsilosis*: a new emerging threat in the fungi arena. Front Fungal Biol. 2022. 10.3389/ffunb.2022.1010782. PMC10512360 37746202

[10] Habibzadeh A, Lankarani KB, Farjam M, et al. Prevalence of fungal drug resistance in COVID-19 infection: a global meta-analysis. Curr Fungal Infect Rep. 2022;16(4):154–64. 10.1007/s12281-022-00439-9. PMC9376562 35990407

[11] Thomaz DY, De Almeida JN, Lima GME, et al. An azole-resistant *Candida parapsilosis* outbreak: clonal persistence in the intensive care unit of a Brazilian teaching hospital. Front Microbiol. 2018. 10.3389/fmicb.2018.02997. PMC6290035 30568646

[12] Hare RK, Arastehfar A, Rosendahl S, et al. Candidemia among hospitalized pediatric patients caused by several clonal lineages of *Candida parapsilosis*. J Fungi. 2022;8(2):1–11. 10.3390/jof8020183. PMC8880282 35205937

[13] Corzo-Leon DE, Peacock M, Rodriguez-Zulueta P, Salazar-Tamayo GJ, MacCallum DM. General hospital outbreak of invasive candidiasis due to azole-resistant *Candida parapsilosis* associated with an Erg11 Y132F mutation. Med Mycol. 2021;59(7):664–71. 10.1093/mmy/myaa098. PMC8257413 33305313

[14] Thomaz DY, de Almeida JN, Sejas ONE, et al. Environmental clonal spread of azole-resistant *Candida parapsilosis* with erg11-y132f mutation causing a large candidemia outbreak in a Brazilian cancer referral center. J Fungi. 2021. 10.3390/jof7040259. PMC8066986 33808442

[15] Li Y, Hind C, Furner-Pardoe J, Sutton JM, Rahman KM. Understanding the mechanisms of resistance to azole antifungals in *Candida* species. JAC Antimicrob Resist. 2025. 10.1093/jacamr/dlaf106. PMC12205959 40583995

[16] Brassington PJT, Klefisch FR, Graf B, et al. Genomic reconstruction of an azole-resistant *Candida parapsilosis* outbreak and the creation of a multi-locus sequence typing scheme: a retrospective observational and genomic epidemiology study. Lancet Microbe. 2025. 10.1016/j.lanmic.2024.07.012. 39557054

[17] Thomaz DY, Del Negro GMB, Ribeiro LB, et al. A Brazilian inter-hospital candidemia outbreak caused by fluconazole-resistant *Candida parapsilosis* in the COVID-19 era. J Fungi. 2022. 10.3390/jof8020100. PMC8874954 35205855

[18] The European Committee on Antimicrobial Susceptibility Testing. Breakpoint tables for interpretation of MICs for antifungal agents, version 11.0, 2024. http://www.eucast.org/astoffungi/clinicalbreakpointsforantifungals/.

[19] Pulcrano G, Roscetto E, Iula VD, Panellis D, Rossano F, Catania MR. MALDI-TOF mass spectrometry and microsatellite markers to evaluate *Candida parapsilosis* transmission in neonatal intensive care units. Eur J Clin Microbiol Infect Dis. 2012;31(11):2919–28. 10.1007/s10096-012-1642-6. 22644055

[20] Escribano P, Rodríguez-Créixems M, Sánchez-Carrillo C, Muñoz P, Bouza E, Guinea J. Endemic genotypes of *Candida albicans* causing fungemia are frequent in the hospital. J Clin Microbiol. 2013;51(7):2118–23. 10.1128/JCM.00516-13. PMC3697706 23616451

[21] Misas E, Witt LS, Farley MM, et al. Molecular and epidemiological investigation of fluconazole-resistant *Candida parapsilosis* - Georgia, United States, 2021. Open Forum Infect Dis. 2021. 10.1093/ofid/ofae264. PMC11146139 38835496

[22] Lockhart SR, Etienne KA, Vallabhaneni S, et al. Simultaneous emergence of multidrug-resistant *Candida auris* on 3 continents confirmed by whole-genome sequencing and epidemiological analyses. Clin Infect Dis. 2017;64(2):134–40. 10.1093/cid/ciw691. PMC5215215 27988485

[23] Gouveia-Eufrasio L, de Freitas GJC, da Silva DL, et al. Acetylation-mediated fluconazole inactivation: a novel antifungal resistance mechanism. Drug Resist Updat. 2026. 10.1016/j.drup.2026.101359. 41581456

[24] Semet C, Kazak E, Ak-Aksoy S, et al. Silent persistence: molecular evidence of clonal transmission in fluconazole-resistant *Candida parapsilosis* hospital outbreaks over decades. J Fungi. 2025;11(11):802. 10.3390/jof11110802. PMC12653143 41295182

[25] Arastehfar A, Daneshnia F, Hilmioğlu-Polat S, et al. First report of candidemia clonal outbreak caused by emerging fluconazole-resistant *Candida parapsilosis* isolates harboring Y132F and/or Y132F+K143R in Turkey. Antimicrob Agents Chemother. 2020;64(10):e01001-20. 10.1128/AAC.01001-20. PMC7508617 32690638

[26] Singh A, Singh PK, de Groot T, et al. Emergence of clonal fluconazole-resistant *Candida parapsilosis* clinical isolates in a multicentre laboratory-based surveillance study in India. J Antimicrob Chemother. 2019. 10.1093/jac/dkz029. 30753525

[27] Martini C, Torelli R, de Groot T, De Carolis E, Morandotti GA, De Angelis G, et al. Prevalence and clonal distribution of azole-resistant *Candida parapsilosis* isolates causing bloodstream infections in a large Italian hospital. Front Cell Infect Microbiol. 2020;10:232. 10.3389/fcimb.2020.00232. PMC7261875 32523896

[28] Trevijano-Contador N, Torres-Cano A, Carballo-González C, et al. Global emergence of resistance to fluconazole and voriconazole in *Candida parapsilosis* in tertiary hospitals in Spain during the COVID-19 pandemic. Open Forum Infect Dis. 2022;9(11):605. 10.1093/ofid/ofac605. PMC9709632 36467290

[29] Branco J, Silva AP, Silva RM, et al. Fluconazole and voriconazole resistance in *Candida parapsilosis* is conferred by gain-of-function mutations in MRR1 transcription factor gene. Antimicrob Agents Chemother. 2015. 10.1128/aac.00842-15. PMC4576118 26248365

[30] Doorley LA, Rybak JM, Berkow EL, et al. *Candida parapsilosis* Mdr1B and Cdr1B are drivers of Mrr1-mediated clinical fluconazole resistance. Antimicrob Agents Chemother. 2022;66(7):e0028922. 10.1128/aac.00289-22. PMC9295576 35699442

[31] Fekkar A, Blaize M, Bouglé A, et al. Hospital outbreak of fluconazole-resistant *Candida parapsilosis*: arguments for clonal transmission and long-term persistence. Antimicrob Agents Chemother. 2023;95(5):e02036-20. 10.1128/AAC.02036-20. PMC8092880 33593841

